# APASL clinical practice recommendation: how to treat HCV-infected patients with renal impairment?

**DOI:** 10.1007/s12072-018-9915-5

**Published:** 2018-12-11

**Authors:** Tatsuo Kanda, George K. K. Lau, Lai Wei, Mitsuhiko Moriyama, Ming-Lung Yu, Wang-Long Chuang, Alaaeldin Ibrahim, Cosmas Rinaldi Adithya Lesmana, Jose Sollano, Manoj Kumar, Ankur Jindal, Barjesh Chander Sharma, Saeed S. Hamid, A. Kadir Dokmeci, Mamun-Al-Mahtab, Geofferey W. McCaughan, Jafri Wasim, Darrell H. G. Crawford, Jia-Horng Kao, Osamu Yokosuka, Shiv Kumar Sarin, Masao Omata

**Affiliations:** 1 Division of Gastroenterology and Hepatology, Department of Medicine, Nihon University School of Medicine, Tokyo, Japan; 2 Humanity and Health Medical Center, Hong Kong, SAR China; 3 Peking University People’s Hospital, Peking University Hepatology Institute, Beijing, China; 4 Kaohsiung Municipal Ta-Tung Hospital, Kaohsiung, Taiwan; 5 Hepatobiliary Division, Department of Internal Medicine, Kaohsiung Medical University Hospital, Kaohsiung Medical University, Kaohsiung, Taiwan; 6 GI/Liver Division, Department of Internal Medicine, University of Benha, Benha, Egypt; 7 Digestive Disease and GI Oncology Centre, Medistra Hospital, Jakarta, Indonesia; 8 Hepatobiliary Division, Department of Internal Medicine, Dr. Cipto Mangunkusumo Hospital, Universitas Indonesia, Jakarta, Indonesia; 9 University Santo Tomas Hospital, Manila, Philippines; 10 Department of Hepatology, Institute of Liver and Biliary Sciences, New Delhi, India; 11 Department of Gastroenterology, G.B. Pant Hospital, New Delhi, India; 12 Department of Medicine, Aga Khan University and Hospital, Stadium Road, Karachi, 74800 Pakistan; 13 Department of Gastroenterology, Ankara University School of Medicine, Ankara, Turkey; 14 Department of Hepatology, Bangabandhu Sheikh Mujib Medical University, Dhaka, 1000 Bangladesh; 15 Royal Prince Alfred Hospital, Centenary Institute, University of Sydney, Sydney, Australia; 16 School of Medicine, University of Queensland, Woolloongabba, QLD 4102 Australia; 17 National Taiwan University College of Medicine and National Taiwan University Hospital, Taipei, Taiwan; 18 Graduate School of Medicine, Chiba University, Chiba, Japan; 19 Yamanashi Prefectural Central Hospital, 1-1-1 Fujimi, Kofu-shi, Yamanashi 400-8506 Japan; 20 The University of Tokyo, 7-3-1 Hongo, Bunkyo-ku, Tokyo, 113-8655 Japan

**Keywords:** HCV, Renal impairment, DAA, SVR, Hemodialysis, Guideline

## Abstract

Chronic hepatitis C virus (HCV) infection is common among patients with chronic kidney disease (CKD) and those on hemodialysis due to nosocomial infections and past blood transfusions. While a majority of HCV-infected patients with end-stage renal disease are asymptomatic, some may ultimately experience decompensated liver diseases and hepatocellular carcinoma. Administration of a combination of elbasvir/grazoprevir for 12 weeks leads to high sustained virologic response (SVR) rates in patients with HCV genotypes (GTs) 1a, 1b or 4 and stage 4 or 5 CKD. Furthermore, a combination of glecaprevir/pibrentasvir for 8–16 weeks also results in high SVR rates in patients with all HCV GTs and stage 4 or 5 CKD. However, these regimens are contraindicated in the presence of advanced decompensated cirrhosis. Although sofosbuvir and/or ribavirin are not generally recommended for HCV-infected patients with severe renal impairment, sofosbuvir-based regimens may be appropriate for those with mild renal impairment. To eliminate HCV worldwide, HCV-infected patients with renal impairment should be treated with interferon-free therapies.

**Electronic supplementary material:**

The online version of this article (10.1007/s12072-018-9915-5) contains supplementary material, which is available to authorized users.

## Introduction

Hepatitis C virus (HCV) infection causes liver diseases including chronic hepatitis, cirrhosis and hepatocellular carcinoma (HCC), as well as, extrahepatic manifestations [1–3]. Chronic kidney disease (CKD) is one such extrahepatic manifestation of HCV infection [3]. HCV infection also causes cryoglobulinemia and cryoglobulin deposits on vascular endothelium, triggering vasculitis in organs such as the kidneys [4]. HCV-related nephropathy is a type I membranoproliferative glomerulonephritis, commonly in the context of type II mixed cryoglobulinemia [5].

Patients with end-stage renal diseases are at high risk for HCV infection due to the need of repeated blood transfusions for anemia (prior to the availability of blood product screening for HCV infection) [6] and hemodialysis (up to ~ 91%) [7–9]. Chronic HCV infection also seems to be associated with a higher prevalence of CKD and shorter renal survival, compared with controls [10]. Interferon-based treatments have been associated with severe adverse events for HCV-infected patients with CKD [2].

After the approval of direct-acting antiviral (DAA), treatment initiation in HCV-infected patients with CKD seemed to be less likely in HCV genotype (GT) 2 or 3 and those with diabetes, cardiovascular disease, alcohol abuse or dependence or cirrhosis at baseline [11]. Because HCV NS5B inhibitor sofosbuvir, which also is effective for HCV GT 2 or 3, has not been recommended for patients with severe renal impairment, and HCV-infected patients with more advanced CKD and other complications are less likely to receive treatment for HCV.

Strategies are needed to improve the treatment for HCV-infected patients with renal impairment. In the present article, we discuss recent strategies with interferon-free treatments, which could result in higher sustained virologic response (SVR) rates, for HCV-infected patients with CKD.

## Treatment for patients with HCV-related liver diseases, CKD stage 5 with/without hemodialysis and having renal transplant prospect

In general, treatment in setting of kidney transplantation, timing of HCV treatment may be before kidney transplant and if therapy needed after kidney transplantation, careful attention should be paid to drug interactions with immunosuppressive agents. Treatment options for hepatitis C in presence of CKD also depend upon the possibility of renal transplant in near future as well as the severity of underlying liver disease. In patients with compensated HCV-related liver disease, CKD stage 5 with/without hemodialysis and having renal transplant prospect, it is advisable to initiate antiviral therapy post renal transplantation.

**Fig. 1:**
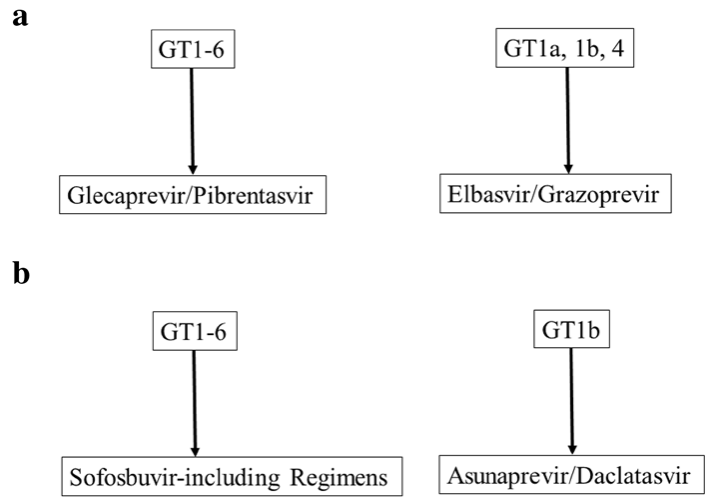
APASL recommendation for the treatment regimens of patients with HCV and severe impaired renal function. **a** For glecaprevir/pibrentasvir and/or elbasvir/grazoprevir-affordable/available countries. **b** For glecaprevir/pibrentasvir and/or elbasvir/grazoprevir-unaffordable/unavailable countries. Although several studies demonstrated that high SVR rate by asunaprevir and daclatasvir in patients with HCV genotype 1b (GT1b) with resistance-associated substitution (RAS) and renal impairment, this combination should be avoided in patients with HCV GT1b with RAS

CKD patients with HCV-related advanced cirrhosis [patients with clinical decompensation and/or hepatic venous pressure gradient (HVPG) > 10 mmHg] require combined liver–kidney transplantation, which is often not feasible, especially in living donor-related liver transplant (LRLT) settings and considering no other options, these patients should be treated with sofosbuvir-based regimens under close monitoring. This regimen may not be optimal for severe CKD which the guideline does not refute but it may be also too late for the underlying liver disease. Thus, this approach may be reserved for situations where also LRLT is not an option. However, when LRLT is an option such patients may be rescued by LRLT and post-transplant could have their advanced CKD managed by hemodialysis while receiving DAA treatment for non-cirrhotic post-transplant chronic hepatitis C. Although the previous study [12] has demonstrated that DAA treatment is safe and effective at post-kidney transplantation, it may be better for kidney transplantation candidates with HCV-related decompensated cirrhosis and CKD stage 4 or 5 to be treated with sofosbuvir-based regimens under close monitoring before transplantation. Because the patient receives a new kidney, any harm of a sofosbuvir based regimen on kidney function may be less important for kidney transplantation candidate. This approach may be an option in kidney transplantation candidates but not in liver transplantation candidates. Further study will be needed (Fig. 1).

Real-life data from the ongoing HCV-TARGET study have also demonstrated the efficacy of DAA therapy in patients with kidney transplant and in those with dual liver–kidney transplant. Various regimens were used, including sofosbuvir/ledipasvir with or without ribavirin (85%); sofosbuvir plus daclatasvir with or without ribavirin (9%); and ombitasvir/paritaprevir/ritonavir plus dasabuvir with or without ribavirin (6%). The SVR12 rate was 94.6% in those with kidney transplant and 90.9% in dual liver–kidney transplant recipients [13].

## Classification of chronic kidney disease (CKD)

The definition of CKD includes all cases with markers of kidney damage [albuminuria (albumin creatine ratio > 3 mg/mmol), hematuria (of presumed or confirmed renal origin), electrolyte abnormalities due to tubular disorders, renal histological abnormalities, structural abnormalities detected by imaging or a history of kidney transplantation] or those with an eGFR < 60 ml/min/1.73 m^2^ on at least 2 occasions 90 days apart with or without markers of kidney damage [14]. In general, some patients who have undergone a kidney transplant or have CKD treated with immunosuppressive therapy and/or have anemia, are not always amenable to treatment with some direct-acting antivirals (DAAs) for HCV which is excreted through kidney [2, 15] (Table 1). It may be more appropriate to avoid the use of sofosbuvir or ribavirin in patients with GFR < 30 ml/min/1.73 m^2^ or patients with a GFR < 50 ml/min/1.73 m^2^, respectively. Thus, careful attention should be paid to the selection of DAAs for HCV-infected patients with renal impairment. We describe two regimens that are relatively safe and recommended for patients with CKD stages 4 and 5.

**Table 1:** Selection of DAA regimens based on renal function

DAAs	Target of DAAs			Metabolism	
	NS3/4A	NS5A	NS5B	Hepatic metabolism/metabolites	Renal excretion
Telaprevir	Yes	–	–	Yes	–
Boceprevir	Yes	–	–	Yes	–
Simeprevir	Yes	–	–	Yes	–
Grazoprevir	Yes	–	–	Yes	–
Asunaprevir	Yes	–	–	Yes	–
Paritaprevir	Yes	–	–	Yes	–
Glecaprevir	Yes	–	–	Yes	–
Voxilaprevir	Yes	–	–	Yes	–
Daclatasvir	–	Yes	–	Yes	–
Ledipasvir	–	Yes	–	Yes	–
Ombitasvir	–	Yes	–	Yes	–
Elbasvir	–	Yes	–	Yes	–
Pibrentasvir	–	Yes	–	Yes	–
Velpatasvir	–	Yes	–	Yes	–
Sofosbuvir^a^	–	–	Yes	–	Yes
Beclabuvir	–	–	Yes	Yes	–
Ribavirin^b^	–	–	–	–	Yes

*DAAs*, direct-acting antiviral agents

^a^Better to use in patients with eGFR ≥ 30 ml/min/1.73 m^2^

^b^Better to use in patients with eGFR ≥ 50 ml/min/1.73 m^2^ and hemoglobin ≥ 12 g/dl

## Selection of DAAs in HCV-infected patients with severe renal impairment

### Treatment with a 12-week combination of grazoprevir/elbasvir for patients with HCV GT1a, GT1b or GT4

The C-SURFFER study included a total of 224 HCV GT1 and stages 4 and 5 CKD patients: 111 and 113 patients who belong to immediate treatment and deferred treatment groups with HCV NS3/4A protease inhibitor grazoprevir (100 mg daily)/HCV NS5A inhibitor elbasvir (50 mg daily) for 12 weeks, respectively [16]. Of these patients, 179 (76%) were hemodialysis-dependent, 122 (52%) were infected with HCV GT1a, 189 (80%) were HCV treatment-naïve, 14 (6%) had cirrhosis, and 108 (46%) were African American. SVR rates in the immediate treatment and deferred treatment groups with grazoprevir/elbasvir were 99% (115/116) and 98% (97/99), respectively [16]. Thus, grazoprevir/elbasvir could lead to higher SVR rates in patients with HCV GT1 and stages 4 and 5 CKD [16, 17]. In the C-SURFFER study [16], the most common adverse events were headache, nausea and fatigue. Cardiac serious events (one cardiac arrest, one cardiomyopathy and three myocardial infarction) were reported in five cases [16]. Other serious adverse events reported in more than one patient were: hypotension, pneumonia, upper gastrointestinal hemorrhage, and aortic aneurysm [16].

Asselah et al. [18] demonstrated that, among HCV GT4-infected patients treated with 12 or 16 weeks of grazoprevir/elbasvir with or without ribavirin, the SVR12 rates in treatment-naïve and treatment-experienced (previously failed pegylated interferon-based treatment) were 96% (107/111) and 89% (39/44), respectively. Although HCV NS5A resistance-associated substitutions (RASs) emerged at virologic failure, baseline HCV NS5A RASs did not impact the SVR 12 rates in the 12 weeks arm of grazoprevir/elbasvir [18].

In patients with HCV GT1a, 1b or 4 and stage 4 or 5 CKD, the use of elbasvir and grazoprevir without ribavirin are recommended by the American Association for the Study of Liver Diseases (AASLD) and Infectious Diseases Society of America (IDSA) [19].

### Treatment with a pangenotypic combination of glecaprevir/pibrentasvir for 8–16 weeks

Gane et al. [20] reported that the HCV NS3/4A protease inhibitor glecaprevir combined with the HCV NS5A inhibitor pibrentasvir could result in 98% (102/104) SVR rates in patients with HCV and severe renal impairment [CKD stage 4, 13% (14 patients); CKD stage 5, 87% (90 patients); and/or hemodialysis, 82% (85 patients)]. Their study included a total of 104 patients [male, 76% (79 patients); mean age, 57 years; and mean eGFR in patients not undergoing hemodialysis, 20.6 ml/min/1.73 m^2^]. The numbers of HCV GT1a, GT1b, GT1 other subgenotypes, GT2, GT3, GT4, GT5 and GT6 were 23, 29, 2, 17, 11, 20, 1 and 1, respectively. The number of treatment-naïve and cirrhotic patients were 58% (60 patients) and 19% (20 patients), respectively. Common adverse events were pruritus (20%), fatigue (14%) and nausea (12%) [20]. Serious adverse events were observed in 24% (25/104). One patient with hemodialysis and hypertension had a cerebral hemorrhage at 2 weeks post-end of treatments (EOT). One patient discontinued treatment at 2 weeks because of non-serious adverse events of diarrhea. Three additional patients discontinued treatments: one at week 8 due to pruritus; one at week 10 due to pulmonary edema, hypertensive cardiomyopathy and congestive heart failure; and one at week 12 due to a hypertensive crisis [20].

The combination of glecaprevir (300 mg daily)/pibrentasvir (120 mg daily) was shown to lead to high SVR rates in Japanese HCV GT1 or GT2-infected patients with severe renal impairment (eGFR < 30 ml/min/1.73 m^2^) [21].

The use of glecaprevir/pibrentasvir without ribavirin is also recommended by the American Association for the Study of Liver Diseases (AASLD) and Infectious Diseases Society of America (IDSA) for the treatment of patients with HCV GT1, GT2, GT3, GT4, GT5, or GT6 and severe renal impairment [19].

### Treatment with a 24-week combination of asunaprevir/daclatasvir without ribavirin for HCV GT1b patients

The combination of HCV NS3/4A protease inhibitor asunaprevir (200 mg daily) and HCV NS5A inhibitor daclatasvir (60 mg daily) for 24 weeks resulted in 100% (16/16) and 100% (8/8) SVR rates in stages 3b, 4 and 5 CKD and HCV GT1b-patients, respectively [22]. This treatment combination also reportedly led to 96% (20/21) and 100% (28/28) SVR rates [23, 24]. Thus, a 24-week combination of asunaprevir/daclatasvir without ribavirin may be a treatment option for patients with HCV GT1b and severe renal impairment [25], although this regimen requires measurement of HCV GTs and HCV NS5A RASs before treatment [26].

## Selection of DAAs in HCV-infected patients with mild renal impairment

### Sofosbuvir-based regimens in patients with HCV and renal impairment

Sofosbuvir and/or ribavirin are excreted through the kidney, therefore, in general, it may be more appropriate to avoid the use of sofosbuvir or ribavirin in patients with CKD stage 3a, 3b, 4 or 5. However, it has been reported that HCV NS5B inhibitor sofosbuvir-based regimens have been used for HCV-infected patients with severe renal impairment (Table 2) [27–29]. Patients with CKD stages 3b/4/5 (eGFR < 30 ml/min/1.73 m^2^) or on hemodialysis seemed to tolerate sofosbuvir-based regimens well. Although sofosbuvir-based regimens could lead to higher SVR rates, sofosbuvir is converted into inactive metabolites and safe and effective doses of sofosbuvir in patients with an eGFR < 30 ml/min have not been established [19]. Taneja et al. [27] reported that low-dose sofosbuvir and full-dose HCV NS5A inhibitor daclatasvir are safe and effective in treating patients with HCV and CKD stages 3b/4/5. Japanese study [30] demonstrated that SVR rates were 97.0, 97.1 and 94.7% and incidence rates of adverse events were 0, 0.5 and 3.0% in sofosbuvir/ledipasvir-treated HCV GT1b-patients with CKD stages 1, 2 and 3, respectively. Although the half-daily dose of sofosbuvir and daclatasvir could also lead to 90.2% SVR rates in 41 patients with HCV GTs 1 and 3 patients and none had a relapse, 2 patients discontinued, and 3 patients died during treatment [31]. Sofosbuvir should be used with caution in patients with severe renal impairment (eGFR < 30 ml/min/1.73 m^2^) without other treatment options, as the pharmacokinetics and safety of sofosbuvir derived metabolites under these circumstances are still being ascertained [32]. In general, sofosbuvir (400 mg daily) may be recommended for patients with CKD stages 1/2/3 (recommendation B-2 [15]). Generic sofosbuvir and branded sofosbuvir play a role in the elimination of HCV worldwide [28].

**Table 2:** Sofosbuvir-based regimens in patients with HCV and renal impairment

Ref.	GTs	No. of patients	CKD	Treatment-naïve/cirrhosis	Regimens	SVR12
Taneja et al. [27]	GT1, 42 (65%); GT2, 1 (1%); GT3, 22 (34%)	65	eGFR < 30; HD, 54 (83%)	55 (85%)/21 (32%)	12- or 24-week of 200 mg SOF/60 mg DCV	100% (65/65)
Kumar et al. [28]	GT1a, 17; GT1b, 1; GT3a, 7; GT3b, 1	26	CKD stage 4,5 or HD (eGFR < 30)	19 (73%)/22 (85%)	24-Week of generic SOF/RBV	100% (26/26)
Kumar et al. [28]	GT1a, 22; GT1b, 4	26	CKD stage 4,5 or HD (eGFR < 30)	23 (89%)/20 (77%)	12-Week of generic SOF/LDV	100% (26/26)
Kumar et al. [28]	GT3a, 17; GT3b, 2	19	CKD stage 4,5 or HD (eGFR < 30)	16 (84%)/12 (63%)	12-Week of generic SOF/DCV	100% (19/19)
Sho et al. [29]	GT2	40	CKD stage 3a/3b	29 (73%)/NA	12-Week of SOF/RBV	90% (36/40)

*Ref.* reference, *GTs* genotypes, *No* number, *CKD* chronic kidney disease, *HD* hemodialysis, *eGFR* estimated glomerular filtration rate, *SVR12* sustained virological response at 12 weeks, *SOF* sofosbuvir, *DCV* daclatasvir, *LDV* ledipasvir, *RBV* ribavirin, *NA* not available

## Drug–drug interactions (DDIs) in patients with renal impairment

The rate of DDIs in patients who suffer from CKD is significant [33]. Comorbidity and polypharmacy are common in CKD-patients [34]. Most of DDAs have DDIs with some cardiovascular drugs [35]. Some statins are not recommended for concomitant use with glecaprevir/pibrentasvir treatment [36]. There is a high rate of clinically significant DDIs between DAAs and anti-epileptic medications [37]. During treatment with DAAs, which are even metabolized by the liver, careful management of DDIs should be required in HCV-infected patients with CKD, who are using cardiovascular drugs, statins or anti-epileptic medications. Frequently encountered DDIs in the setting of CKD are shown in Suppl. Table 2.

## Conclusion

HCV infection is usually asymptomatic in patients with end-stage renal disease [38]; however, it may lead to decompensated liver diseases and HCC. In some patients with HCV infection and severe renal impairment, treatment with DAAs is discontinued due to renal dysfunction [39]. For patients with CKD stages 4/5, hemodialysis should be prepared if renal function worsens and treatment with a DAA combination is initiated. In some patients undergoing hemodialysis, treatment with a DAA combination may be safer than those with CKD stages 4/5.

Recommendations for the treatment of patients with HCV and severe renal impairment are shown in Tables 3 and 4. A combination of elbasvir/grazoprevir is recommended for patients with HCV GT1a, 1b or 4 and stage 4 or 5 CKD [40]. A combination of glecaprevir/pibrentasvir is recommended for patients with HCV all GTs and stage 4 or 5 CKD (Table 3). Sofosbuvir-based regimens may be suitable for HCV-infected patients with mild renal impairment [28]. It has been reported that a higher frequency of anemia, worsening renal dysfunction and more severe adverse events were observed in patients with low baseline renal function [41]. To eliminate HCV worldwide, HCV-infected patients with renal impairment should be treated with these combination therapies.

**Table 3:** APASL recommendation for the treatment regimens of patients with HCV and severe impaired renal function

HCV GTs	Regimens	Treatment duration (weeks)	Grading of evidence and recommendations (disease status)
GT1a, GT1b, GT4	Elbasvir (50 mg daily)/grazoprevir (100 mg daily)	12	A-1 (CKD 3b/4/5 or hemodialysis)
All GTs	Glecaprevir (300 mg daily)/pibrentasvir (120 mg daily)	8–16	A-1 (CKD 3b/4/5 or hemodialysis)
GT1b	Daclatasvir (60 mg daily)/asunaprevir (200 mg daily)	24	B-2 (CKD 3b/4/5 or hemodialysis)
All GTs	Sofosbuvir (400 mg daily)/daclatasvir (60 mg daily) under close monitoring	12	B-2 (CKD 3b/4/5 or hemodialysis)
GT1	Sofosbuvir (400 mg daily)/ledipasvir (90 mg daily) under close monitoring	12	B-2 (CKD 3b/4/5 or hemodialysis)

Grading of evidence and recommendations are shown in Suppl. Table 1

*GTs* genotypes

**Table 4:** Treatment regimens for patients with hepatitis C virus infection and severe renal impairment: APASL recommendation (this article), compared with those of EASL [32] or AASLD-IDSA [19]

Regimens	APASL	EASL	AASLD-IDSA
Elbasvir/grazoprevir	A-1 [CKD 3b/4/5 or hemodialysis (GT1a, 1b, 4)]	A-1 [CKD 3b/4/5 or hemodialysis (GT1b)]	B-1 [CKD 3b/4/5 or hemodialysis (GT1a, 1b, 4)]
Glecaprevir/pibrentasvir	A-1 [CKD 3b/4/5 or hemodialysis (all GTs)]	A-1 [CKD 3b/4/5 or hemodialysis (all GTs)]	B-1 [CKD 3b/4/5 or hemodialysis (all GTs)]
Daclatasvir/asunaprevir	B-2 [CKD 3b/4/5 or hemodialysis (GT1b)]	No description	No description
Sofosbuvir-based regimens	B-2 [CKD 4/5 or hemodialysis (all GTs)]	B-1 (alternative treatment)	Not recommendation and need close monitoring [41]
Ritonavir-boosted paritaprevir/ombitasvir/dasabuvir	No description	A-1 [CKD 3b/4/5 or hemodialysis (GT1b)]	No description

Grading of evidence and recommendations are shown in Suppl. Table 1

*GTs* genotypes, *CKD* chronic kidney disease

## Electronic supplementary material

Below is the link to the electronic supplementary material.

Supplementary material 1 (DOCX 22 kb)

## Abbreviations

HCV: Hepatitis C virus
GT: Genotype
HCC: Hepatocellular carcinoma
DAAs: Direct-acting antivirals
SVR: Sustained virological response
CKD: Chronic kidney disease
