# Supplementary material for: APASL clinical practice recommendation: how to treat HCV-infected patients with renal impairment?
Source: Hepatol Int. 2018 Dec 11;13(2):103–9. doi: 10.1007/s12072-018-9915-5 (PMC6418053; doi:10.1007/s12072-018-9915-5)
Supplement: Supplementary file 1 — Supplementary material 1 (DOCX 22 kb) [file 12072_2018_9915_MOESM1_ESM.docx]

**Suppl. Table 1.** Grading of evidence and recommendations (adapted from the GRADE system) [1, 2].

| **Grading of evidence** | **Notes** | **Symbol** |
| --- | --- | --- |
| High quality | Further research is very unlikely to change our confidence in the estimate of effect. | A |
| Moderate quality | Further research is likely to have an important impact on our confidence in the estimate of effect and may change the estimate. | B |
| Low or very low quality | Further research is very likely to have an important impact on our confidence in the estimate of effect and is likely to change the estimate. Any estimate of effect is uncertain. | C |
| **Grading of recommendation** | **Notes** | **Symbol** |
| Strong recommendation warranted | Factors influencing the strength of the recommendation included the quality of the evidence, presumed patient important outcomes, and cost. | 1 |
| Weaker recommendation | Variability in preferences and values or more uncertainty make it more likely that a weak recommendation is warranted. Recommendation is made with less certainty; higher cost or resource consumption. | 2 |

Ref

1. Guyatt GH, Oxman AD, Vist GE, Kunz R, Falck-Ytter Y, Alonso-Coello P, et al. GRADE: an emerging consensus on rating quality of evidence and strength of recommendations. BMJ 2008;336:924–926

2. Schunemann HJ, Oxman AD, Brozek J, Glasziou P, Jaeschke R, Vist GE, et al. Grading quality of evidence and strength of recommendations for diagnostic tests and strategies. BMJ 2008;336:1106–1110

**Suppl. Table 2.** Potential drug-drug interactions (DDIs) between direct-acting antivirals (DAAs) against hepatitis C virus and representative other drugs in the setting of chronic kidney disease [3-5].

| **Regimens** | **Representative drugs inducing DDIs with DAAs** |
| --- | --- |
| Elbasvir/grazoprevir | **Antiretroviral drugs:** Efavirenz, Etravirine, Nevirapine, Atazanavir/ritonavir, Atazanavir/cobicistat, Darunavir/ritonavir, Darunavir/cobicistat, Lopinavir/ritonavir, Elvitegravir/cobicistat/emtricitabine/tenofovir disoproxil fumarate, Elvitegravir/cobicistat/emtricitabine/tenofovir alafenamide. **Illicit/recreational drugs:** Fentanyl, Gamma-hydroxybutyrate, Oxycodone. **Statins:** Atorvastatin, Fluvastatin, Gemfibrozil, Lovastatin, Rosuvastatin, Simvastatin. **Central nervous system drugs:** Aripiprazole, Quetiapine. **Cardiovascular drugs:** Amiodarone. **Immunosuppressants:** Cyclosporine, Sirolimus, Tacrolimus. **Antiplatelets and anticoagulants**: Dabigatran, Ticagrelor, Rivaroxaban, Apixiban, Edoxaban, Warfarin. |
| Glecaprevir/pibrentasvir | **Antiretroviral drugs:** Efavirenz, Etravirine, Nevirapine, Atazanavir/ritonavir, Atazanavir/cobicistat, Darunavir/ritonavir, Darunavir/cobicistat, Lopinavir/ritonavir. **Illicit/recreational drugs:** Fentanyl, Gamma-hydroxybutyrate, Oxycodone. **Statins:** Atorvastatin, Ezetimibe, Fluvastatin, Gemfibrozil, Lovastatin, Pitavastatin, Pravastatin, Rosuvastatin, Simvastatin. **Central nervous system drugs:** Aripiprazole, Clozapine, Paliperidone, Quetiapine. **Cardiovascular drugs:** Amiodarone, Digoxin, Carvedilol, Diltiazem, Aliskiren, Enalapril. **Immunosuppressants:** Cyclosporine, Sirolimus, Tacrolimus. **Antiplatelets and anticoagulants**: Dabigatran, Ticagrelor, Rivaroxaban, Apixiban, Edoxaban, Warfarin. |
| Daclatasvir/asunaprevir | **Antiretroviral drugs:** Efavirenz, Etravirine, Nevirapine, Atazanavir; atazanavir/ritonavir, Fosamprenavir, Saquinavir, Elvitegravir/cobicistat/emtricitabine/tenofovir disoproxil fumarate. **Illicit/recreational drugs:** Cannabis, Cocaine, Diazepam, Gamma-hydroxybutyrate, Ketamine, Phencyclidine (PCP). **Statins:** Atorvastatin, Fluvastatin, Lovastatin, Pitavastatin, Pravastatin, Rosuvastatin, Simvastatin. **Central nervous system drugs:** Trazodone, Aripiprazole, Clozapine, Haloperidol, Quetiapine, Risperidone. **Cardiovascular drugs:** Amiodarone, Digoxin, Bisoprolol, Amlodipine, Diltiazem, Nifedipine, Aliskiren. **Immunosuppressants:** Cyclosporine, Everolimus, Tacrolimus**. Antiplatelets and anticoagulants**: Clopidogrel, Dabigatran. |
| Sofosbuvir-based regimens | **Antiretroviral drugs:** Tenofovir disoproxil fumarate, Efavirenz, Etravirine, Nevirapine, Sirolimus, Elvitegravir/cobicistat/emtricitabine/tenofovir disoproxil fumarate. **Statins:** Atorvastatin, Fluvastatin, Lovastatin, Pitavastatin, Pravastatin, Rosuvastatin, Simvastatin. **Central nervous system drugs:** Paliperidone. **Cardiovascular drugs:** Amiodarone, Digoxin, Carvedilol, Amlodipine, Diltiazem, Aliskiren. **Antiplatelets and anticoagulants**: Dabigatran, Ticagrelor, Rivaroxaban, Apixiban, Edoxaban, Warfarin |
| Ritonavir-boosted paritaprevir/ombitasvir/dasabuvir | **Antiretroviral drugs:** Tenofovir alafenamide, Efavirenz, Etravirine, Nevirapine, Rilpivirine, Atazanavir/ritonavir, Atazanavir/cobicistat, Darunavir/ritonavir, Darunavir/cobicistat, Lopinavir/ritonavir, Elvitegravir/cobicistat/emtricitabine/tenofovir disoproxil fumarate, Elvitegravir/cobicistat/emtricitabine/tenofovir alafenamide. **Illicit/recreational drugs:** Amphetamine, Cannabis, Cocaine, Diamophine, Diazepam, Gamma-hydroxybutyrate, Ketamine, MDMA (ecstasy), Mefedrone, Methamphetamine, Oxycodone, Phencyclidine (PCP). **Statins:** Atorvastatin, Ezetimibe, Fluvastatin, Gemfibrozil, Lovastatin, Pitavastatin, Pravastatin, Rosuvastatin, Simvastatin. **Central nervous system drugs:** Amitriptyline, Sertraline, Trazodone, Venlafaxine, Aripiprazole, Chlorpromazine, Clozapine, Flupentixol, Haloperidol, Olanzapine, Quetiapine, Risperidone, Zuclopentixol. **Cardiovascular drugs:** Amiodarone, Digoxin, Vernakalant, Flecainide, Bisoprolol, Carvedilol, Amlodipine, Diltiazem, Nifedipine, Aliskiren, Doxazosin, Enalapril. **Immunosuppressants:** Cyclosporine, Etanercept, Mycophenolate, Sirolimus, Tacrolimus. **Antiplatelets and anticoagulants**: Clopidogrel, Dabigatran, Ticagrelor, Rivaroxaban, Apixiban, Edoxaban, Warfarin. |

**Ref**

1. European Association for Study of Liver. EASL Recommendations on Treatment of Hepatitis C 2015. J Hepatol 2015;63:199–236.
2. European Association for the Study of the Liver. EASL Recommendations on Treatment of Hepatitis C 2018. J Hepatol 2018;69:461–511.
3. University of Liverpool. HEP Drug Interactions. www.hep-druginteractions.org, accessed on 8/7/2018.
